# Cervical cancer prevention in Indonesia: An updated clinical impact, cost-effectiveness and budget impact analysis

**DOI:** 10.1371/journal.pone.0230359

**Published:** 2020-03-23

**Authors:** Didik Setiawan, Andrijono, Sri Rezeki Hadinegoro, Hashta Meyta, R. Vensya Sitohang, Gertrudis Tandy, Dyah Aryani Perwitasari, Maarten J. Postma

**Affiliations:** 1 Faculty of Pharmacy, Universitas Muhammadiyah Purwokerto, Banyumas, Indonesia; 2 Center for Health Economic Studies, Universitas Muhammadiyah Purwokerto, Banyumas, Indonesia; 3 Division of Oncology, Department Obstetric and Gynecologic, Cipto Mangunkumo Hospital, Jakarta, Indonesia; 4 Faculty of Medicine, University of Indonesia, Jakarta, Indonesia; 5 Department of Child Health, Faculty of Medicine, University of Indonesia, Indonesia; 6 Indonesian Immunization Technical Advisory Group (Indonesia-NTAG), Indonesia; 7 Directorate General of Diseases Prevention and Control (P2P) Ministry of Health, Indonesia; 8 Faculty of Pharmacy, Universitas Ahmad Dahlan, Yogyakarta, Indonesia; 9 Unit of Pharmacoepidemilogy & Pharmacoeconomics (PE2), Department of Pharmacy, University of Groningen, Groningen, The Netherlands; Massachusetts General Hospital, UNITED STATES

## Abstract

**Introduction:**

The clinical and economic impact of cervical cancer consistently become a serious burden for all countries, including Indonesia. The implementation of HPV vaccination policy for a big country such as Indonesia requires a strong commitment from several decision-makers. The aim of this study was to provide a comprehensive description on cost-effectiveness and the budget-impact of HPV vaccination policy in Indonesia.

**Method:**

A cohort Markov model was used to evaluate the cost and the clinical impact of HPV vaccination for 10 years old girls in Indonesia. The researchers consider two doses of all three available HPV vaccines adjusted with the HPV infection profilewith 95% vaccination coverage to estimate the national cervical cancer incidence and mortality. The Budget impact analysis explores three different scenarios covering (1) Two districts per year expansion, (2) oneprovince per year expansion and (3) achieving the National Immunization Program in 2024.

**Results:**

Upon fully vaccinating almost 2.3 million 10-year-old girls, 34,723; 43,414; and 51,522 cervical cancer cases were prevented by Quadrivalent, Bivalent and Nonavalent vaccines, consecutively. Furthermore, the highest (591 cases) and lowest (399 cases) mortality were prevented by Nonavalent and Quadrivalent vaccines, respectively. Most of the vaccines were considerably cost-effective and only the Bivalent vaccine with the GAVI/UNICEF price which will be considered a cost-saving strategy.To provide national coverage of HPV vaccination in Indonesia, the government has to provide an annual budget of about US$49 million and US$22 million using the government contract price and GAVI/UNICEF price, respectively.

**Conclusion:**

HPV vaccination shows a cost-effective strategy and the budget required to provide this policy is considerably affordable for Indonesia.

## Introduction

The high clinical and economic burden of cervical cancer has been experienced by many countries in the world, particularly developing countries such as Indonesia. The latest global cancer survey showed that cervical cancer is the second-highest incidence and mortality rates among women with the age-standardized incidence and mortality rate of 13.1 and 6.9 per 100,000, respectively [1]. In 2011, the Indonesian government spent about US$2.5 million for cervical cancer treatment and it was only for the low and middle social economic population which are covered by Social health Insurance (Jamkesmas)[2]. Currently, in the Universal Health Coverage era, which has been implemented since 2014, the clinical and economic burden of cervical cancer are potentially much higher since cancer treatment is fully covered by the government.

Although the cause of cervical cancer and its prevention strategy has been clearly explained and recommended by several bodies including WHO, the comprehensive implementation of the recommendation, covering HPV vaccination, cervical screening, and cancer treatment as the primary, secondary and tertiary intervention, consecutively, faces some substantial milestone due to several issues for example parent’s acceptance, availability of skilled human resources, and most importantly, the required budget.However, several health economic studies showed that the implementation of HPV vaccination and cervical screening are cost-effective strategies in most countries in the world [3–5]. Moreover, the implementation of those strategies will also substantially save the national account of Indonesia that should be spent on cervical cancer treatment [6,7].

The current prices of HPV vaccines are considerably expensive. In addition, since Indonesia entered the GAVI accelerated transition phase in 2016, Indonesia is currently not eligible for co-financing policy implementation scheme where vaccines is funded by both Indonesia and GAVI. Therefore, self-financing of HPV vaccination policy by the government of Indonesia requires a huge amount of national budget since the government has to provide about five million (two doses x 2.5 million girls on age 10) doses annually. On the other hand, the pilot project that has been initiated since 2016 in Jakarta Province and it was followed by several regions/regencies, such as Yogyakarta, Bali, Makassar, and Manado, in the next following years. The results of this pilot was promising since the vaccination coverage was mostly higher than 90%.

Since these prevention programs require strong political commitment from several stakeholders, including the ministry of health, ministry of planning and most importantly, ministry of finance, an evidence-based policy recommendation is required in order to provide a comprehensive clinical and economic impact of HPV vaccination policy in Indonesia. Therefore, this study objectives was to provide not only the updated cost-effectiveness analysis of cervical cancer prevention using HPV vaccines in Indonesia but also its required budget in order to provide this particular strategies nationwide.

## Methods

### Model structure and current situation in Indonesia

A cohort Markov model, that has been developed previously[6], was updated using the latest- and country-specific-data to project the epidemiological profile of cervical cancer in Indonesia. The model followed a cohort of 2,277,200 of 10-year-old girls, which are the main target of HPV vaccination policy in 2019, and this cohort was followed until the population is 85 years old. The epidemiological profile of cervical cancer was modeled from the current Global Cancer Statistics 2018 [8] and also considering the cervical screening policy that has been formally implemented since 2015 in Indonesia[9]. In Indonesia, both Visual Inspections using Acetic Acid (VIA) screening and pap smear are available for free for 30–50 years old women in Community Health Care Center (Pusat Kesehatan Masyarakat–Puskesmas). Our base case implemented several cervical cancer screening profiles for Indonesia including 21% of screening coverage, 10% of detection rate [10]and 83% of cryotherapy coverage[11].

### HPV vaccine efficacy and coverage

Vaccine efficacies on preventing cervical cancer incidence were derived from efficacies data and then adjusted with the HPV infection profile in Indonesia[6,12]. Only high-risk HPV (hrHPV) types, that are considered as the main causes of cervical cancer, were included in this study including HPV type 16, 18, 31, 33, 45, 52, and 58 (Table 1) [13]. Although there is only two available HPV vaccine in Indonesian market (Quadrivalent from MSD® and Bivalent HPV vaccine from GSK®), this study also considers Nonavalent HPV vaccine (from MSD®) in order to provide a comprehensive description. In addition, this study also considered all the possible cross-protection properties of HPV vaccines based on several studies[14–16].

**Table 1:** The prevalence of HPV infections and vaccine characteristics.

HPV Types	Prevalence of infection (%)	Vaccine efficacy (%) (b)			Source
	(a)[13]	Bivalent	Quadrivalent	Nonavalent	
16	47	95	95	95	[14,15]
18	20	95	95	95	[14–16]
31	0	79	0	95	[14–16]
33	30	56	0	95	[14–16]
45	6	76	0	95	[14–16]
52	6	0	0	95	[15]
58	0	0	0	95	[15]
Vaccine effectiveness (axb)		0,79	0,64	0,94	[6,12]

### Cost, utility and discount rate

Although Indonesia has been included as GAVI Accelerated Transition Countries since 2016, GAVI vaccine prices are still applicable for Indonesia as long as the procurement is implemented through UNICEF until 2025. GAVI Vaccine price for Bivalent, Quadrivalent and Nonavalent vaccines are US$4.6, US$4.5, and US$6.9, respectively[17]. Currently, Indonesia has a contract price for the Quadrivalent vaccine (US$11.79) if the government procure through the pharmaceutical Industry. Since there is no official information about contract price for the other two vaccines, we calculate the assumed vaccine price for Bivalent and Nonavalent based on the contract price of Quadrivalent and weighted with the GAVI vaccine price comparison. The vaccination cost will not only consider the vaccine price but also the shipping cost, insurance and the handling fee including custom clearance, warehouse storage, transportation, and labeling particularly for HPV vaccine that procured through UNICEF (Table 2). While the vaccine price from the pharmaceutical company did not consider the handling cost since it is included in the vaccine price.

**Table 2:** The vaccination cost component of HPV vaccine.

Cost component	GAVI/UNICEF Price*	Government contract price*
Vaccine price	4.50	11.62
Shipping cost	0.16	0.00
Handling fee	0.41	
Insurance	0.23	

*Vaccine price using Gardasil^®^ price for the budget impact analysis.

The initial and recurrent cervical cancer costs were adopted from previous studies [6] and screening and cryotherapy costs were adopted from the national tariffs for cervical cancer prevention policy[18]. All the cost information was converted and updated to 2018 US$ using conversion rate and inflation rate [19].

The utility values for the susceptible population was derived from a study by Purba *et*. *Al*., (2018) about the Quality of Life of the Indonesian General Population [20]. The utility value for the cervical cancer population was adopted from a study by Setiawan *et*.*al*., (2018)evaluating the Health-Related Quality of Life of HPV-related cancer patients including cervical cancer [21] and the death population is valued by the utility of 0 (zero). According to Indonesian HTA body, both cost and utility were discounted by 3% annually[22].

### Clinical, economic and health-economic outcomes

The clinical impacts of all three HPV vaccines on the Indonesian population were presented as the incidence- and mortality-rate reduction in the cohort. While the incremental cost and utility were presented as the differences in cost and utility between each vaccine compared to the current situation in Indonesia. Moreover, the Incremental Cost-Effectiveness Ratio (ICER) is calculated by dividing the incremental cost with the incremental effectiveness, utility or Quality Adjusted Life Years (QALYs) is used in this study.

### Sensitivity analysis

Univariate sensitivity analysis was performed in order to investigate the robustness of the ICER. This analysis was performed by implementing the minimum and maximum values of several variables; including vaccine coverage (99.70% - 99.90%), cervical cancer treatment cost (US$US$1,215 –US$3,018), discount rate for cost and utility (0% - 5%), vaccine cost (US$8.84 –US$14.74), utility for susceptible population (0.80–1.00), and (quadrivalent) vaccine effectiveness (0.48–0.80).

### Budget impact analysis

The HPV vaccination program has been implemented in one province (DKI Jakarta) and other five regions (Surabaya, Makassar, Manado, Gunung Kidul and Kulon Progo) in 2018. In 2019, the expansion program has been executed to add three additional districts of Yogyakarta (Bantul, Sleman, and Yogyakarta City) in order to provide HPV vaccination for all districts in Yogyakarta Province. The impacts of the HPV vaccination policy for 2020 to 2024 were described comprehensively according to the Ministry of Health plan and proposed scenario. The budget impact was estimated according to the projected population of Indonesia 2010–2025 from the Indonesian Central Bureau of Statistics [23] and the province-based incidence rate of cervical cancer.

The target population was divided into (1) new population, representing the population who get the first dose of vaccination, and (2) recurrence population, representing the population who get the second dose of vaccination. Based on these criteria, the required budget for HPV vaccination was calculated annually. In this BIA, we also calculated the potential cost reduction, potential incidence, and mortality reduction by running the scenario of HPV vaccination implementation for every 10 years old cohort annually from 2020 to 2024 and compared it with the no-vaccination policy in Indonesia.

There were three scenario of HPV vaccination policy implementation: (1) introduction to two district per year based on the province with high cervical cancer incidence, (2) introduction to 1 province per year based on the cervical cancer incidence rate, (3) introduction to several province excluding the province with Regional Gross Domestic Product (GDP) equal or higher than GDP of Bali province that has to be self-funded. The third scenario was chosen since the province of Bali introduced an HPV vaccination program in one of the districts in the region in 2017. According to this finding, we assume that if a region has sufficient budget, synergistic collaboration between local and central government will speed up the National Immunization Program for HPV in Indonesia.

## Results

### The clinical outcomes of HPV vaccination in Indonesia

**Fig 1:**
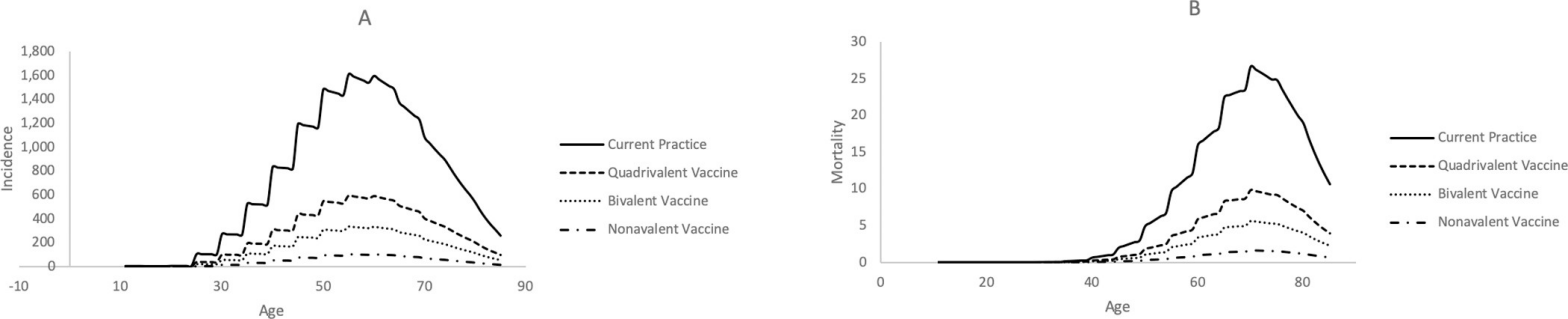
The incidence (A) and mortality (B) of cervical cancer in Indonesian women population.

HPV vaccines show a promising clinical impact on the Indonesian women population (Fig 1). The highest reduction in both cervical cancer incidence and mortality is generated by the Nonavalent vaccine followed by the Bivalent vaccine and the Quadrivalent vaccine. This results showed that the impact of total coverage on hrHPV, which is possessed by Nonavalent vaccine, and cross-protection, which is possessed by Bivalent vaccine, apparently increase the efficacy of the vaccine and therefore reduce more cervical cancer incidence and mortality.

The profile of cervical cancer incidence and mortality reduction caused by all three different HPV vaccines showed a comparable patterns. While Nonavalent vaccines potentially eliminates cervical cancer by providing 93.78%, reduction on cervical cancer incidence, currently available vaccines in Indonesia will only reduce the incidence up to 63% and 79% for Quadrivalent and Bivalent, respectively.

### The cost-effectiveness of HPV vaccination in Indonesia

All three vaccines generate additional discounted QALYs in the Indonesian women population due to cervical cancer incidence and mortality reduction. Under the GAVI-price, the implementation of HPV vaccination policy using the Bivalent vaccine generates lower discounted-total cost compared to the current situation (-US$3.48M), while the other two vaccines require an additional cost of US$0.95M and US$2.45M for Quadrivalent and Nonavalent vaccine, consecutively. Furthermore, only the bivalent vaccine is considered as a cost-saving vaccination program while the other two vaccines show a small value of ICER (Table 3).

**Table 3:** The discounted cost, QALYs and ICER of three different vaccines and two different prices in Indonesia.

Strategies	Discounted Cost	Discounted QALYs	Δ Discounted Cost	Δ Discounted QALYs	ICER
Current situation	30,909,793	56,269,764	Reference	Reference	Reference
***GAVI/UNICEF Price***					
Quadrivalent vaccine	31,858,072	56,313,177	948,279	43,414	22
Bivalent vaccine	27,433,330	56,323,993	-3,476,463	54,229	-64
Nonavalent vaccine	33,360,609	56,334,064	2,450,816	64,300	38
***Government Contract Price***					
Quadrivalent vaccine	65,059,648	56,313,177	34,149,855	43,414	787
Bivalent vaccine	61,372,718	56,323,993	30,462,925	54,229	562
Nonavalent vaccine	84,269,692	56,334,064	53,359,899	64,300	830

If the Government contract price is implemented in the country, a substantial increase of discounted-costs are required (>US$30M) for all three vaccines with the highest addition is required by the Nonavalent vaccine (US$53.36M). However, since there is a huge amount of benefits represented by QALYs value, the ICER generated by all three vaccines are considerably highly-cost-effective (US$562-US$830/QALYs)[24].

**Fig 2:**
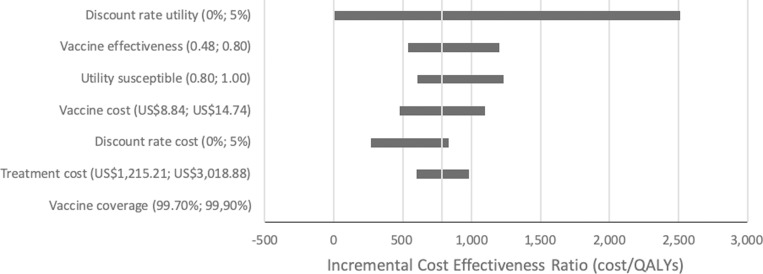
Univariate sensitivity analysis for HPV vaccination.

One-way sensitivity analysis was used to evaluate the impact of each parameter on the ICER (Fig 2). Our study shows that the ICER was most sensitive to discount rate of utility followed by vaccine effectiveness and utility of susceptible population. However, the implementation of HPV vaccination in Indonesia remained cost-effective under all investigated scenarios.

### Budget impact of HPV vaccination program in Indonesia

To describe the budget impact of the HPV vaccination implementation program in Indonesia, we performed a budget impact analysis (BIA) for the next five years of implementation (2024). The first scenario is intended to introduce HPV vaccination program for two districts per year based on the province incidence rate in Indonesia (Table 4). This scenario will cover for less than 10% (230,060 of 2,337,000 girls) of the targeted population in 2024. Since the implementation scenario requires small annual budget for both GAVI/UNICEF price (USD 1,494,000 in 2019 and up to USD 2,398,000 in 2024) and government contract price (USD 3,267,000 in 2019 and up to USD 5,247,000 in 2024), the saving that will be generated by this vaccination is less than USD 600,000 annually. Furthermore, the clinical impact of the HPV vaccination program using this scenario will generate cervical cancer incidence and mortality reduction for about 6.18% and 6.20%, consecutively.

**Table 4:** The targeted population, budget impact and potential cost reduction on cervical cancer treatment, incidence and mortality cases as the results of the implementation of HPV vaccination program on two regions per year in Indonesia.

Years of implementation	2019	2020	2021	2022	2023	2024
Target population	149,920	162,677	173,171	212,935	221,466	230,060
New Population	18,677	12,757	10,494	15,254	8,531	8,594
Recurrence	131,243	149,920	162,677	197,681	212,935	221,466
Vaccine cost (USD 000)						
GAVI/UNICEF price Total	1,494	1,660	1,784	2,262	2,307	2,398
Additional cost	99	68	56	162	45	46
Recurrence cost	1,394	1,593	1,728	2,100	2,262	2,353
Govt. price Total	3,267	3,632	3,903	4,949	5,048	5,247
Additional cost	217	148	121	355	99	100
Recurrence cost	3,050	3,484	3,781	4,594	4,949	5,147
Potential cost reduction (USD 000)	344	373	397	489	509	528
Potential reduction (%)						
Incidence	2.272 (4.14)	2.465 (4.41)	2.624 (4.60)	3.228 (5.67)	3.357 (5.93)	2.487 (6.18)
Mortality	26 (4.15)	28 (4.42)	30 (4.61)	37 (5.69)	39 (5.94)	40 (6.20)

Another plan on the HPV vaccination expansion plan in Indonesia is to add one province per year according to the cervical cancer incidence rate(Table 5). This implementation plan will cover about 38% of the targeted population (887,600 of 2,337,000 girls) in 2024. Compared to the first scenario, the expansion of one province per year will increase the required budget up to USD 8.3 million and USD 18.0 million using GAVI/UNICEF price and Government contract price in 2024, respectively. The benefits of HPV vaccination in Indonesia will also higher than the first scenario since the potential annual saving will be about USD 2 million for the 2024 cohort population. Finally, the cervical cancer incidence and mortality cases of the cohort in 2024 will decrease up to 24.12% and 24.18%, respectively.

**Table 5:** The targeted population, budget impact and potential cost reduction on cervical cancer treatment, incidence and mortality cases as the results of the implementation of HPV vaccination program on one province per year in Indonesia.

Years of implementation	2019	2020	2021	2022	2023	2024
Target population	360,759	796,422	819,200	830,700	855,100	887,600
New Population	113,405	307,722	21,100	13,200	31,500	40,200
Recurrence	247,354	488,700	198,100	817,500	823,600	847,600
Vaccine cost (USD 000)						
GAVI/UNICEF price Total	1,916	4,230	7,635	7,769	7,940	8,246
Additional cost	602	1,635	112	70	167	214
Recurrence cost	1,314	2,595	7,523	7,699	7,773	8,033
Govt. price Total	4,192	9,573	16,702	16,995	17,369	18,040
Additional cost	1,318	3,576	245	153	366	467
Recurrence cost	2,874	5,997	16.457	16,842	17,003	17,573
Potential cost reduction (USD 000)	831	1,845	1,898	1,925	1,982	2,059
Potential reduction						
Incidence	5,471 (9.79)	12,099 (21.64)	12,455 (22.26)	12,620 (22.57)	12,992 (23.24)	13,488 (24.12)
Mortality	63 (9.81)	139 (21.69)	143 (22.31)	145 (22.63)	149 (23.29)	155 (24.18)

The synergistic role between central and local governments will possibly enhance the achievement of the National Immunization Program (NIP) in Indonesia (Table 6). While some districts in Bali showed the capability of the self-funding HPV vaccination program. Our third scenario considers the GDP of Bali as a threshold and another province that has GDP equal or higher than Bali will be encouraged to self-funding the HPV vaccination program. Furthermore, the central government budget will be allocated for regions that have a lower GDP than Bali.

**Table 6:** The targeted population, budget impact and potential cost reduction on cervical cancer treatment, incidence and mortality cases as the results of the National Immunization Program in 2024 in Indonesia.

Years of implementation	2019	2020	2021	2022	2023	2024
Target population	149,920	476,010	1,043,216	1,370,816	1,895,116	2,327,916
New Population	18,677	326,656	554,206	334,300	531,000	440,400
Recurrence	131,243	149,354	489,010	1,036,516	1,364,116	1,887,516
Vaccine cost (USD 000)						
GAVI/UNICEF price Total	1,493	3,322	8,139	12,787	17,312	22,391
Additional cost	99	1,735	2,944	1,776	2,821	2,339
Recurrence cost	1,394	1,587	5,195	11,012	14,492	20,052
Govt. price Total	3,267	7,267	17,804	27,973	37,871	48,982
Additional cost	217	3,796	6,440	3,885	6,170	5,117
Recurrence cost	3,050	3,471	11,364	24,088	31,701	43,865
Potential cost reduction (USD 000)	344	1,098	2,424	3,200	4,457	5,508
Potential reduction						
Incidence	2,272 (4.14)	7,222 (12.92)	15,861 (27.78)	20,868 (36.68)	28,906 (51.02)	35,566 (63.07)
Mortality	26 (4.15)	83 (12.95)	182 (27.85)	240 (36.76)	332 (51.10)	408 (63.14)

HPV vaccination coverage will reach almost 100% (2,327,916 of 2,337,000 girls) coverage in 2024 using this scenario. However, the required budget substantially increases up to US$22.3M and US$48.9M using GAVI/UNICEF and government contract price, consecutively. The estimated clinical benefits from this strategy will reduce both the incidence and mortality of about 63%.

## Discussion

In this study, three different expansion scenarios, (1) two districts per year, (2) one province per year, and (3) achieving National Immunization Programin 2024, were simulated in order to describe the impact of these strategies on the national budget, potential cost of treatment reduction, incidence rate and mortality rate. In this budget impact analysis, two different prices, GAVI or UNICEF price, and the government contract price were considered since Indonesia is still eligible to get the vaccines using the GAVI price if the procurement processes are done through UNICEF until 2025.

Generally, higher vaccination coverage will generate more benefits for both Indonesian governments and the population since there will be fewer incidence and death due to cervical cancer and therefore cost of cervical cancer treatment is also reduced substantially and surely, the higher vaccination coverage will generate a substantial required budget. The further question is about how much the Indonesian government will increase the allocation of their current health budget for the HPV vaccination program since the required budget in order to provide a nationwide HPV vaccination program would be about US$22.4 and US$49 Million using GAVI and contract price, respectively in 2024. This value is considerably high since the projected annual budget of vaccination in 2019 is about US$200 Million.

Additionally, this study shows that the provision of HPV vaccines on top of the current situation, VIA screening and pap smear are accessible for free, are considerably cost-saving to cost-effective intervention since the ICER (US$-64 –US$830) lies far below the Indonesian GDP per capita[22,25].These findings are in line with our previous study in Indonesia[6] and most of the cost-effectiveness studies on HPV vaccines in ASEAN countries[26–29].Among three different HPV vaccines, the Bivalent vaccine shows a cost-saving strategy if the Indonesian government procure using GAVI/UNICEF price (ICER of -US$64). Although the Bivalent vaccine price is slightly higher than Quadrivalent vaccine, this result is mainly driven by cross-protection property which is possessed by Bivalent vaccine [30,31]so there will be more cervical cancer incidence and mortality that might be prevented, and furthermore, the cervical cancer treatment cost will be lower than the other vaccines.

One of the limitation on this study is that the use of 1–3 times GDP as a normative threshold or willingness to pay to decide whether an intervention is cost-effective in a country is debatable and sometimes questionable. This statement was initially published by the WHO Commission on Macroeconomics and Health and recommends that an ICER lay beyond three times GDP and one time GDP is cost-effective and highly-cost-effective, respectively [24]. However, many cost-effective interventions, particularly vaccination policy, are not automatically funded or implemented in the country [32]. Therefore, an official threshold in a country and budget impact analysis is required to simplify the decision-making process and make it more transparent.

Formalized WTP or threshold in a country is crucial in order to ensure the transparency of decision making process. For example, using 1 to 3 times GDP-per-capita recommendation, all three vaccines are cost-effective strategies and therefore Bivalent vaccine will be selected since it has the lowest ICER. However, since all those three ICERs are located far below GDP, the decision-maker can choose Nonavalent vaccine since it has the highest effectiveness among all those three vaccines.However, a question about how much budget is required to implement HPV vaccination as a national policy has to be addressed and it has been explained in this study as well.

Although several vaccines (Bivalent and Nonavalent) have considered not only HPV16 and HPV18 but also the most oncogenic types of HPV (HPC31, HPV33, HPV45, HPV52, and HPV58), another limitation of this study is that several serotypes which are commonly found in Indonesia; including HPV39, HPV51, HPV56 and HPV59[33];have not been covered by the current available vaccines[34,35]. This fact could possibly lead to the lower effectiveness of the vaccine among the Indonesia population. However, the mismatch between vaccines types and the actual serotypes in Indonesia provide opportunities for pharmaceutical companies to develop another type of HPV vaccine covering more HPV serotypes to increase the effectiveness of cervical cancer prevention programs in Indonesia.

Another limitation of this study was the use of Static Markov model since it generally underestimates the protection of the HPV vaccine in the population. This model fairly describe the impact of herd immunity of the HPV vaccine and therefore the non-linear relationship between age and HPV vaccine effectiveness was also created. For example, irrespective of the type of HPV, the incidence and mortality rates of cervical cancer have the same trends. Therefore, the best type of model to explain the nature of infection and its prevention is using a dynamic model. The dynamic model could also explain the interaction among the susceptible population, through sexual contact (matrix), and therefore the effectiveness of vaccines on preventing the infection can be explained comprehensively[36,37]. However, since the main results of this study showed that almost all three different vaccines are cost-effective and cost-saving, intervention, the use of static Markov model is considerably sufficient.

Although several limitations, mainly lack of the formalized WTP or threshold in Indonesia, not all serotypes are covered in the study and the use of static Markov Model, are exist in this study, this budget impact analysis of cervical cancer prevention program in Indonesia showed that HPV vaccines are affordable especially when it is procured using GAVE/UNICEF prices.

## Supporting information

S1 Appendix Targeted implementation. (DOCX)
