# Supplementary material for: Cervical cancer prevention in Indonesia: An updated clinical impact, cost-effectiveness and budget impact analysis
Source: PLoS One. 2020 Mar 23;15(3):e0230359. doi: 10.1371/journal.pone.0230359 (PMC7089525; doi:10.1371/journal.pone.0230359)
Supplement: S1 Appendix — (DOCX) [file pone.0230359.s001.docx]

Appendix

Targeted implementation

| Years of Implementation | 2019 | 2020 | 2021 | 2022 | 2023 | 2024 |
| --- | --- | --- | --- | --- | --- | --- |
| Scenario 1  (Two districts per year) | DKI Jakarta (p)  Surabaya  Makassar  Manado  Gn Kidul  Kulon Progo  Bantul*  Sleman*  Yogyakarta* | DKI Jakarta (p)  Surabaya  Makassar  Manado  DIY (p)  Karanganyar*  Sukoharjo* | DKI Jakarta (p)  Surabaya  Makassar  Manado  DIY (p)  Karanganyar  Sukoharjo  Kediri*  Lamongan* | DKI Jakarta (p)  Surabaya  Makassar  Manado  DIY (p)  Karanganyar  Sukoharjo  Kediri  Lamongan  Batam*  Tj Pinang* | DKI Jakarta (p)  Surabaya  Makassar  Manado  DIY (p)  Karanganyar  Sukoharjo  Kediri  Lamongan  Batam  Tj Pinang  Jambi*  Muara Jambi* | DKI Jakarta (p)  Surabaya  Makassar  Manado  DIY (p)  Karanganyar  Sukoharjo  Kediri  Lamongan  Batam  Tj Pinang  Jambi  Muara Jambi  Banjarbaru*  Banjarmasin* |
| Scenario 2  (1 province per year) | DKI Jakarta (p)  Surabaya  Makassar  Manado  Gn Kidul  Kulon Progo  Bantul*  Sleman*  Yogyakarta* | DKI Jakarta  DIY  East Java**  South Sulawesi**  Central Java* | DKI Jakarta  DIY  East Java  South Sulawesi  Central Java  North Sulawesi* | DKI Jakarta  DIY  East Java  South Sulawesi  Central Java  North Sulawesi  Riau Island* | DKI Jakarta  DIY  East Java  South Sulawesi  Central Java  North Sulawesi  Riau Island  Jambi* | DKI Jakarta  DIY  East Java  South Sulawesi  Central Java  North Sulawesi  Riau Island  Jambi  South Kalimantan* |
| Scenario 3  (Reaching NIP in 2024, considering cancer incidence and local GDP for vaccine procurement responsibility) | DKI Jakarta (p)  Surabaya  Makassar  Manado  Gn Kidul  Kulon Progo  Bantul*  Sleman*  Yogyakarta* | DKI Jakarta (p)  Surabaya  Manado  DIY (p)  North Sulawesi**  Central Java*  Riau Island*  Jambi* | DKI Jakarta (p)  DIY (p)  North Sulawesi  Central Java  Riau Island  Jambi  South Kalimantan*  South Sulawesi**  North Maluku*  West Sulawesi*  Bengkulu*  West Sumatera*  Aceh*  Maluku*  East Java* | DKI Jakarta (p)  DIY (p)  North Sulawesi  Central Java  Riau Island  Jambi  South Kalimantan  South Sulawesi  North Maluku  West Sulawesi  Bengkulu  West Sumatera  Aceh  Maluku  East Java  Central Sulawesi*  West Nusa Tenggara*  West Kalimantan*  Southeast Sulawesi*  East Nusa Tenggara*  Papua*  Bali*  Bangka Belitung Island*  East Kalimantan* | DKI Jakarta (p)  DIY (p)  North Sulawesi  Central Java  Riau Island  Jambi  South Kalimantan  South Sulawesi  North Maluku  West Sulawesi  Bengkulu  West Sumatera  Aceh  Maluku  East Java  Central Sulawesi  West Nusa Tenggara  West Kalimantan  Southeast Sulawesi  East Nusa Tenggara  Papua  Bali  Bangka Belitung Island  East Kalimantan  West Java*  Riau*  Central Kalimantan*  West Papua* | DKI Jakarta (p)  DIY (p)  North Sulawesi  Central Java  Riau Island  Jambi  South Kalimantan  South Sulawesi  North Maluku  West Sulawesi  Bengkulu  West Sumatera  Aceh  Maluku  East Java  Central Sulawesi  West Nusa Tenggara  West Kalimantan  Southeast Sulawesi  East Nusa Tenggara  Papua  Bali  Bangka Belitung Island  East Kalimantan  West Java  Riau  Central Kalimantan  West Papua  South Sumatera*  Banten*  Lampung*  Gorontalo*  North Sumatera* |

(p) province; *introduction region; **province with vaccination program in some district previously;
